# Survival analysis after stereotactic ablative radiotherapy for early stage non-small cell lung cancer: a single-institution cohort study

**DOI:** 10.1186/s13014-024-02439-0

**Published:** 2024-04-18

**Authors:** Kamila Resova, Lukas Knybel, Tereza Parackova, Marian Rybar, Karel Cwiertka, Jakub Cvek

**Affiliations:** 1 Dept. of Oncology, University Hospital Ostrava, 17. listopadu 1790, 708 52 Ostrava, Czech Republic; 2 Faculty of Medicine and Dentistry, Palacky University Olomouc, Olomouc, Czech Republic; 3 Faculty of Medicine, University of Ostrava, Ostrava, Czech Republic; 4 Department of Biomedical Technology, Faculty of Biomedical Engineering, Czech Technical University in Prague, Kladno, Czech Republic; 5 Department of Oncology, University Hospital Olomouc, Olomouc, Czech Republic

**Keywords:** SBRT, ES-NSCLC, Overall survival

## Abstract

**Background:**

Stereotactic ablative radiotherapy (SABR) is the standard treatment for medically inoperable early-stage non-small cell lung cancer (ES-NSCLC), but which patients benefit from stereotactic radiotherapy is unclear. The aim of this study was to analyze prognostic factors for early mortality.

**Methods:**

From August 2010 to 2022, 617 patients with medically inoperable, peripheral or central ES-NSCLC were treated with SABR at our institution. We retrospectively evaluated the data from 172 consecutive patients treated from 2018 to 2020 to analyze the prognostic factors associated with overall survival (OS). The biological effective dose was > 100 Gy_10_ in all patients, and 60 Gy was applied in 3–5 fractions for a gross tumor volume (GTV) + 3 mm margin when the tumor diameter was < 1 cm; 30–33 Gy was delivered in one fraction. Real-time tumor tracking or an internal target volume approach was applied in 96% and 4% of cases, respectively. In uni- and multivariate analysis, a Cox model was used for the following variables: ventilation parameter FEV1, histology, age, T stage, central vs. peripheral site, gender, pretreatment PET, biologically effective dose (BED), and age-adjusted Charlson comorbidity index (AACCI).

**Results:**

The median OS was 35.3 months. In univariate analysis, no correlation was found between OS and ventilation parameters, histology, PET, or centrality. Tumor diameter, biological effective dose, gender, and AACCI met the criteria for inclusion in the multivariate analysis. The multivariate model showed that males (HR 1.51, 95% CI 1.01–2.28; *p* = 0.05) and AACCI > 5 (HR 1.56, 95% CI 1.06–2.31; *p* = 0.026) were significant negative prognostic factors of OS. However, the analysis of OS showed that the significant effect of AACCI > 5 was achieved only after 3 years (3-year OS 37% vs. 56%, *p* = 0.021), whereas the OS in one year was similar (1-year OS 83% vs. 86%, *p* = 0.58).

**Conclusion:**

SABR of ES-NSCLC with precise image guidance is feasible for all medically inoperable patients with reasonable performance status. Early deaths were rare in our real-life cohort, and OS is clearly higher than would have been expected after best supportive care.

## Background

Primary lung cancer a common life-threatening malignancy and the main cause of death among all cancers [1]. Stereotactic ablative radiation therapy (SABR) is the standard of care for medically inoperable, early-stage non-small cell lung cancer (ES-NSCLC), as it provides very high local control rates and induces minimal toxicity [2–4]. However, radiation-related decompensation of chronic diseases cannot be excluded. The overall survival (OS) is poorer and more variable for patients with localized NSCLC treated with stereotactic body radiation therapy (SBRT) than for patients undergoing surgery [5]. The ESTRO recommendation considers a short life expectancy as a contraindication for radical treatment [6]. However, predicting poor survival is challenging, and a 6-month interval for death has been used as a benchmark of short-term survival. A systematic review reported a mean survival of 11.94 months for untreated ES-NSCLC [7].

For many years, the presence of significant comorbidity and poor performance status have been considered important independent prognostic factors for survival [8, 9], and many other parameters, such as the Charlson Comorbidity Index (CCI) [10], age-adjusted Charlson Comorbidity Index (AACCI) [11], Cumulative Illness Rating Score (CIRS) [12], sarcopenia [13], smoking status, and/or Global Initiative for Chronic Obstructive Lung Disease (GOLD) score [14], have been proposed as metrics. Age and gender have also been presented as independent prognostic factors [15]. Finally, the survival rates after SABR may be impacted by tumor volume [16], tumor histology [17], pre-SBRT SUV_max_ [18], and lower lobe location [19].

Because of conflicting conclusions, some groups have established nomograms based on the weighted combination of considered parameters [16, 19]. In contrast, other groups have proposed that SBRT should be offered to all patients regardless of their comorbidities unless the performance status of the patients and their comorbidities prevent accurate SBRT planning and delivery [20]. Lastly, to avoid severe toxicity, more fractions and/or lower doses should be used for severe comorbid patients [21].

The primary goal of this retrospective study was the evaluation of early mortality after SABR when the indication is provided by the multidisciplinary team (MDT) based on the repeated evaluation of performance status (PS) without prognostic parameters or nomograms.

## Methods

We retrospectively evaluated the data from consecutive patients with medically inoperable, peripheral or central ES-NSCLC (T1-T2b according to TNM 8th edition) treated with SABR since 2010. Patients with other malignancy or previous lung cancer were excluded from the study. After updating the institutional workflow protocol in 2017, we included 172 patients treated from 2018 to 2020 for further analysis of prognostic factors of OS. All follow-up data were obtained and collected in August 2023. After institutional review board approval, all relevant information, such as ventilation parameters, histology, gender, tumor diameter, tumor location, age, biologically effective dose (BED_10_), and AACCI [22], were reviewed retrospectively. Follow-up radiographic evaluations were performed by the same team of one radiation oncologist and one radiologist. This study was approved by our institutional review board (No. 153/2023).

### Patients

**Fig. 1:**
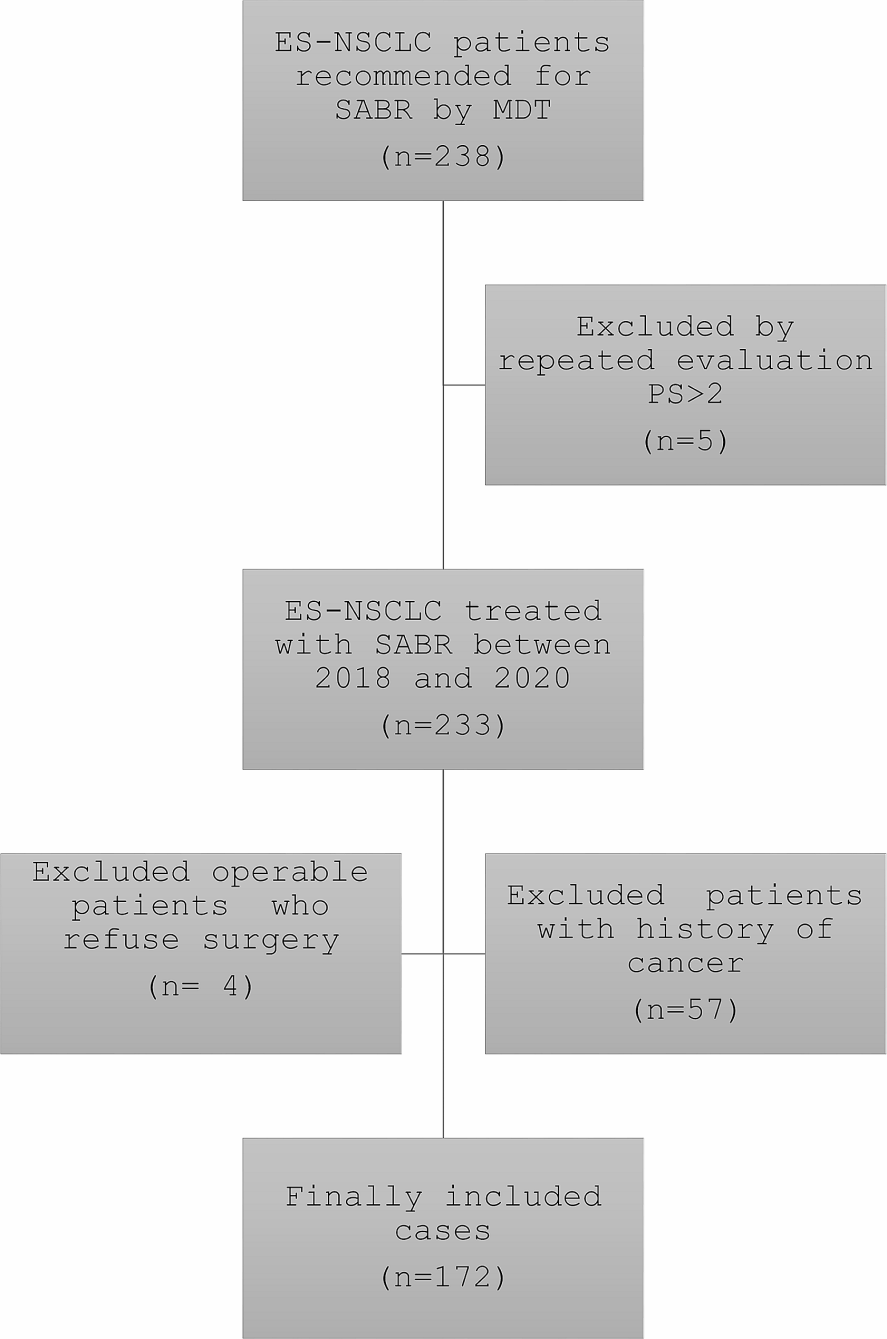
Consort diagram. (In total, 238 patients with ES-NSCLC were recommended for SABR. Patients with PS > 2, operable or with history of cancer were excluded from the study. Finally 172 patients were analyzed)

Patients were deemed medically inoperable in agreement with MDT and eligible for SABR regardless of intercurrent diseases. Cases with interstitial pneumonia were not excluded (4 patients). Only patients with repeated PS worse than 2 were considered for the best supportive care (Fig. 1). Comorbidity was rated using the AACCI, a weighted index of comorbidity for 19 clinical conditions adjusted for age by adding 1 point to the index score for each decade of life over 50. Although the CCI attributes 2 points for “any tumor”, we did not regard primary lung tumor as a comorbidity and did not score it in the tabulation. The classification and staging of chronic obstructive pulmonary disease (COPD) was performed according to GOLD guidelines [23]. FEV1 as a percentage of predicted values (FEV1%) was used as a variable of pulmonary function. Histology was not mandatory in cases of high risk. Table 1 shows the baseline clinical and treatment characteristics.

**Table 1:** Patients and treatment characteristics

Characteristics of 172 Patients		
	No	%
**Age (years)**		
Median (range)	73 (54–92)	
**Sex**		
Male	100	58
Female	72	42
**T stage**		
IA (T1a-c)	110	64
IB (T2a)	39	23
IIA (T2b)	23	13
**Pathologic confirmation**		
Yes ( spino/adeno)	82 (44/70)	48
No	90	52
**PET**		
Yes	105	61
No	67	39
**COPD (GOLD)**		
0	43	25
1	23	13
2	42	24
3	37	22
4	27	16
**FEV1% predicted**		
Median (range**)**	55% (20–137)	
**≥ 50%**	108	63
**< 50%**	64	37
**AACCI**		
Median (range)	5 points (2-10)	
2–5	114	66
6–10	58	34
**Performance Status**		
**0**	45	26
**1**	88	51
**2**	39	23
**Tumor location**		
Central	23	13
Peripheral	149	87
**Dose fractionation**		
60–54 Gy/3fr.	92	53
30–33 Gy/1fr.	19	11
60–55 Gy/5fr.	42	24
50 Gy/5fr.	19	11
**BED**_**10**_ **prescribed**		
Median (range)	151.2 (94–180)	
≥ 151.2 Gy	82	48
< 151.2 Gy	90	52
**BED**_**10**_ **maximum**		
Median (range)	251.6 (135–398)	
≥ 251.6 Gy	75	44
< 251.6 Gy	97	56

For continuous variables, the median and range are given; for categorical variables, the number of patients and percentages are given, AACCI: age-adjusted Charlson comorbidity index; CHOPD: chronic obstructive pulmonary disease; GOLD: Global Initiative for Chronic Obstructive Lung Disease; FEV1: forced expiratory volume in 1 s; BED_10_ biologically effective dose with alpha/beta = 10 Gy

### Treatment

Our technique was described previously [24]. Briefly, withthe patient in a supine position, the chest was imaged in 1 mm-thick native CT scans during expiration breath hold under audio/video couching. The gross tumor volume (GTV) was delineated in the lung window and a 3-mm isotropic margin added. Sequential dose optimization was performed using a Monte Carlo algorithm (22%) or Ray Tracing algorithm (78%). We used several regiments based on tumor location and diameter, mostly 60 Gy in 3–5 fractions. In the case of a tumor diameter < 1 cm, 30–33 Gy was delivered in one fraction. 50 Gy in 5 fractions was the preferred fractionation scheme for centrally located lesions. The dose was prescribed to have a minimum planning target volume (PTV) coverage with the prescribed dose of 95%. The BED_10_ was calculated for the prescribed and maximum doses. The treatment delivery was with the patient free breathing. The CyberKnife system (Accuray, Inc., WI, USA) in conjunction with Synchrony tracking software that enabled real-time respiratory motion tracking was used. The Synchrony system allows detection of the tumor position from 2 orthogonal x-ray images. The correlation model between external diode markers (LED) on the patient’s chest and tumor motion is built before the start of treatment and adapted during treatment with each new x-ray acquisition (the latest 15 data points are used). The motion of the LEDs is tracked with a camera in the treatment room. A robot with a linear accelerator compensates for tumor motion based on the correlation model. If the real-time tracking strategy was not possible, 4D-CT scans were performed with an ITV approach without any gating strategy or fiducial tracking.

### Follow-up

Patients were followed up with clinical examinations in referral hospitals and imaging performed according to their decisions. In general, follow-ups were conducted at 4-month intervals for the first 2 years, and then every 6 months for the next 3 years. PET scans were repeated only in the event of suspected disease relapse in patients who were fit to receive salvage therapy.

### Statistical analysis

We evaluated time-dependent parameters using the Kaplan-Meier method. OS respects all deaths regardless of etiology. A simple univariable Cox model was used for categorical and continuous variables in the univariable analysis. Variables (Table 2) with *p* < 0.25 in the univariable analyses were entered into a multivariable Cox proportionality hazard model with the variables of interest, using *p* < 0.05 to determine an adjusted influence of variables on outcome. The results of the multivariate Cox proportional model were expressed as hazard ratios (HRs) with 95% confidence interval (Cis) and p-values. OS was calculated from the first day of treatment. The proportion of patients who survived at a given timepoint was derived using the Kaplan-Meier method with corresponding two-sided 95% Cis and p-values. Local control was evaluated based on the PET positivity, histology, or start of salvage treatment. Toxicities were graded according to the National Cancer Institute Common Terminology Criteria for Adverse Events version 4.0 [25]. Statistical analyses were performed using R software (Version 4.3.1, R Core Team, Vienna University).

**Table 2:** Univariate and multivariate analyses affecting overall survival

Variables	Univariate analysis			Multivariate analysis		
	HR	95% CI	P value	HR	95% CI	P value
**Age**	0.99	0.97–1.01	0.399			
≥ 78	1					
< 78	1.77	1.10–2.84	0.018			
**Sex**						
Female	1					
Male	1.66	1.11–2.47	0.013	1.51	1.01–2.28	0.047
**AACCI**						
≤ 5	1					
≥ 6	1.67	1.13–2.47	0.010	1.56	1.06–2.31	0.026
**FEV1%**						
≥ 50	1					
< 50	1.06	0.72–1.58	0.762			
**Histology**						
Unproven	1					
Proven	1.16	0.79–1.69	0.457			
Adeno	1.12	0.69–1.81	0.651			
Spino	1.19	0.76–1.87	0.455			
**PET**						
Yes	1					
No	1.04	0.70–1.53	0.860			
**Location**						
Peripheral	1					
Central	1.29	0.72–2.30	0.394			
**Prescribed BED**_**10**_						
≥ 150	1					
< 150	1.31	0.89–1.93	0.168	1.26	0.85–1.88	0.256
**Maximum BED**_**10**_						
≥ 251.6	1					
< 251.6	1.43	0.96–2.11	0.076			
**T stage**						
T1a-c	1					
T2a-b	1.43	0.97–2.11	0.073	1.08	0.90–1.29	0.411

(HR: hazard ratio, CI: confidence interval, AACCI: age-adjusted Charlson comorbidity index, FEV1: forced expiratory volume in 1 s, BED_10_: biologically effective dose with alpha/beta = 10 Gy)

## Results

**Fig. 2:**
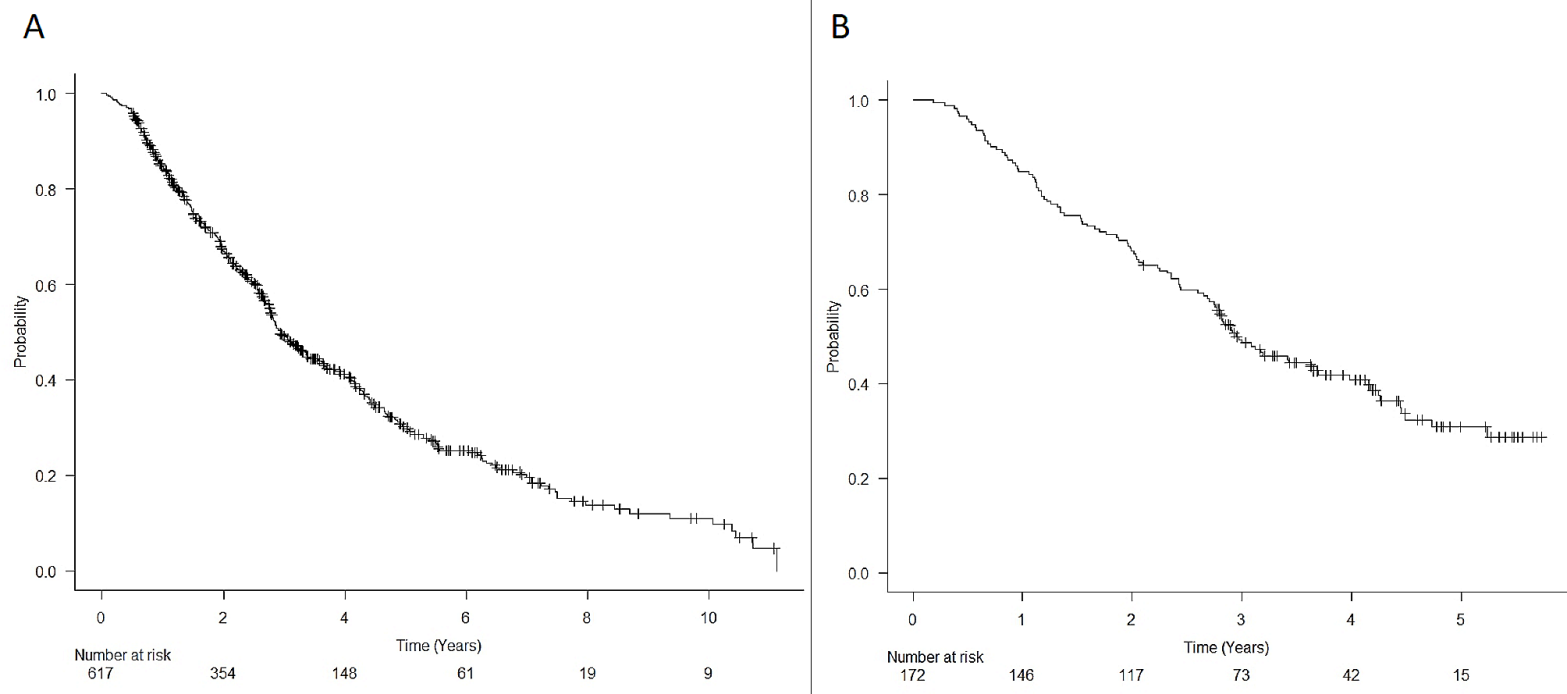
Kaplan-Meier curves showing the of overall survival (OS). Panel **A**: OS of the 617 early-stage lung cancer patients treated with SABR from 2010 to 2022, Panel **B**: OS of the 172 patients treated from 2018 to 2020 used for analysis

From August 2010 to 2022, 617 patients (365 males, 252 females) were treated at our institution. Median survival was 35.2 months, and the 6-month and 1-year OS was 95.9%, and 84.6%, respectively (Fig. 2a).

After updating the treatment protocol in 2017, 172 patients (median age 73 years, range 54–92 years) who underwent SABR from 2018 to 2020 were enrolled in this study. The median survival was 35.3 months, and the 6-month and 1-year OS was 95.9% and 84.9%, respectively (Fig. 2b). Median GTV, dose, number of fractions, and isodose line were 9 ml (range 0.82–87.5 ml), 60 Gy (30–60 Gy), 3 (1-5) and 79% (60-83%), respectively. The real-time tumor tracking strategy was not possible in 7 (4%) patients.

The 1-, 2- and 3-year local control (LC) rates for all patients were 97%, 95% and 90%, respectively. Local relapse was suspicious in 11 patients (6%) and confirmed by histology in 5 (3%). No grade 4 or 5 treatment-related toxicities were reported. Grade 2 toxicity was observed in 12 patients (7%), including radiation pneumonitis, chest wall pain, and esophagitis. Grade 3 toxicity was reported in 4 patients (2%). These adverse events were related to peripheral neuropathy, rib fracture, esophagitis, and hemoptysis. A total of 93 deaths were recorded, 31 of them had disease progression, the remaining 62 patients had no progression or severe toxicity, but the exact cause of death is not available.

**Fig. 3:**
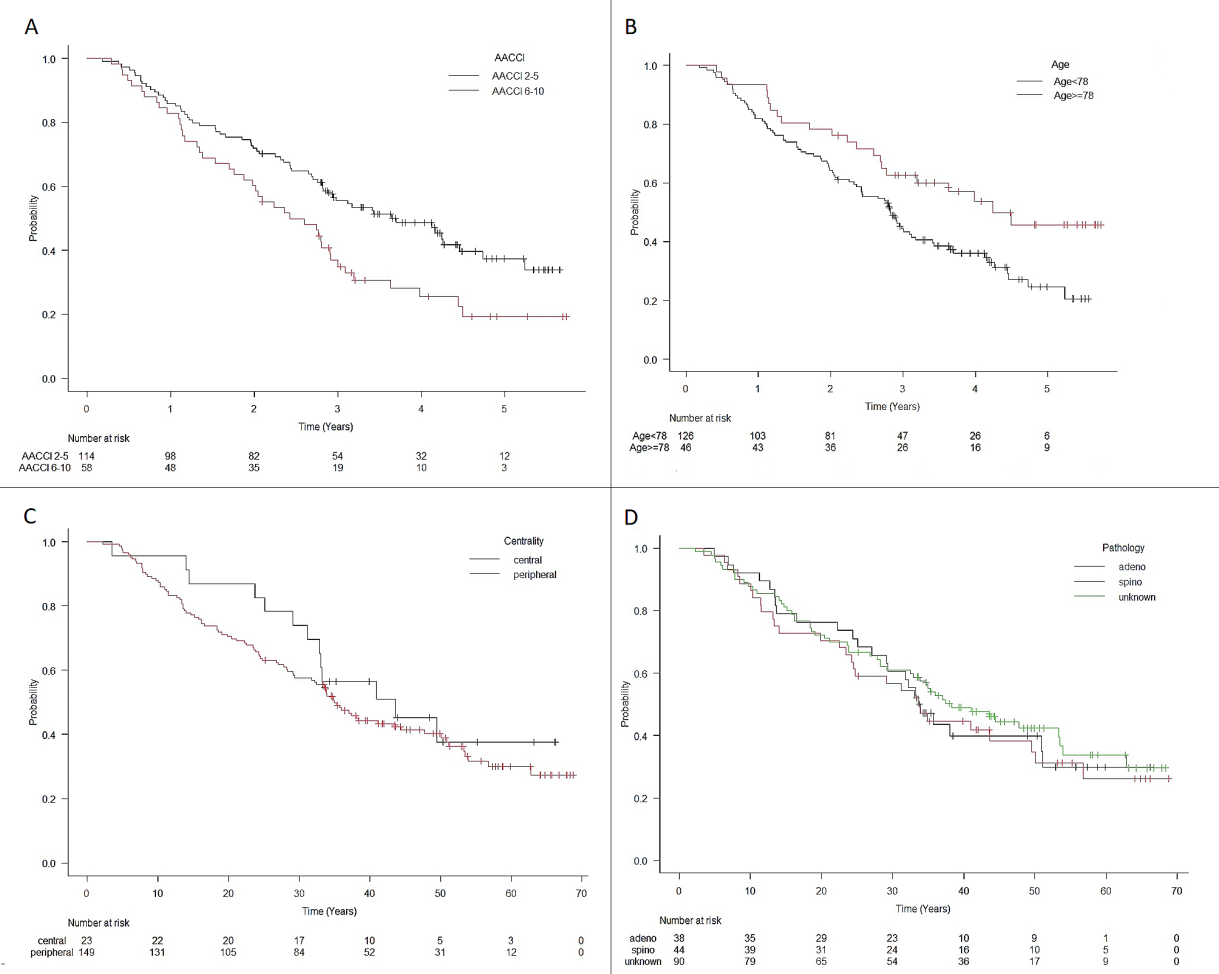
Kaplan-Meier curves of overal survival (OS) with variables from univariate analyses Panel **A**: Age-adjusted Charlson comorbidity index (AACCI 2–5 vs. AACCI 6–10), panel **B**: Age (≥ 78 years vs. < 78 years), panel C: centrality (central vs. peripheral), panel D: pathology (adenocarcinoma vs. squamous cell carcinoma vs. unknown)

**Fig. 4:**
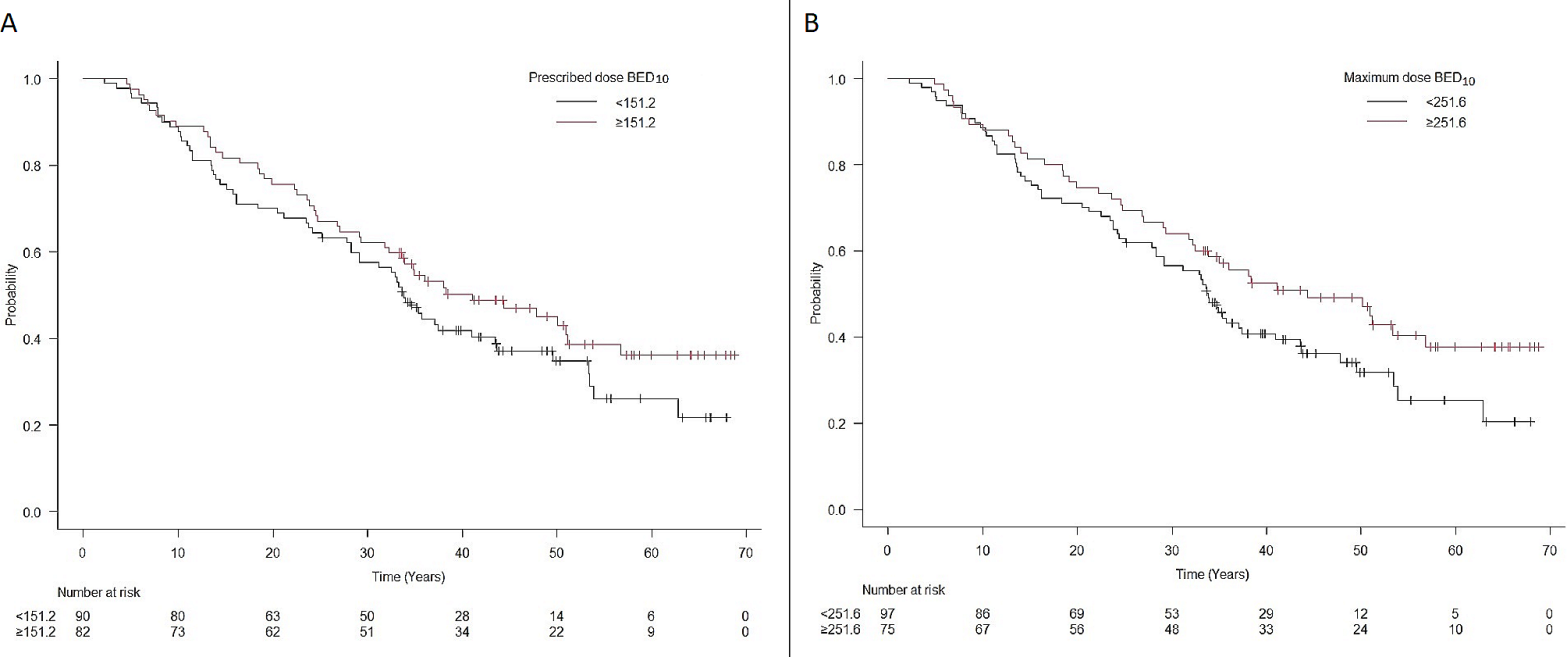
Kaplan-Meier curves of overal survival (OS) stratified by prescribed BED_10_ and maximum BED_10_

Univariate analyses (Table 2) identified the following detrimental variables (Fig. 3a-b) associated with OS: AACCI > 5 (HR 1.67, 95% CI 1.13–2.47; *p* = 0.01), male gender (HR 1.66, 95% CI 1.11–2.47; *p* = 0.013), and age < 78 years (HR 1.77, 95% CI 1.10–2.84; *p* = 0.018). No significant difference was observed in other patient-specific variables (Fig. 3c-d). Prescribed BED_10_ < 151.2 Gy (HR 1.31, 95% CI 0.89–1.93; *p* = 0.17) and maximum BED_10_ < 251.6 Gy (HR 1.43, 95% CI 0.96–2.11; *p* = 0.08) were associated with OS (Fig. 4).

The multivariate model (Table 2) showed that males (HR 1.51, 95% CI 1.01–2.28; *p* = 0.05) and AACCI > 5 (HR 1.56, 95% CI 1.06–2.31; *p* = 0.026) were significant negative prognostic factors of OS. However, the analysis of OS showed that the negative effect of AACCI > 5 was achieved only after 3 years (3-year OS 37% vs. 57%, *p* = 0.021); the differences in OS in 2 years and 1 year were not significant (60% vs. 72%, *p* = 0.12; 86% vs. 83%, *p* = 0.58; Table 3). The prognostic significance of T stage (*p* = 0.41) and prescribed BED (*p* = 0.26) was not significant for OS (Table 2).

**Table 3:** Comparison of short-term and long-term mortality as a function of AACCI (age-adjusted Charlson comorbidity index)

Time	AACCI	Survival %*	95% CI	p
*6 months*	2–5	97,40%	(92,1%; 99,1%)	0,176
	6–10	93,10%	(82,7%; 97,4%)	
*12 months*	2–5	86,00%	(78,1%; 91,2%)	0,579
	6–10	82,80%	(70,3%; 90,3%)	
*18 months*	2–5	78,90%	(70,3%; 85,4%)	0,153
	6–10	69,00%	(55,4%; 79,2%)	
*24 months*	2–5	71,90%	(62,7%; 79,2%)	0,123
	6–10	60,30%	(46,6%;71,6%)	
*36 months*	2–5	55,50%	(45,8%; 64,2%)	0,021
	6–10	36,90%	(24,5%; 49,3%)	

*The proportions of patients who survived at a given timepoint was derived from the Kaplan-Meier method with corresponding two-sided 95% CIs and p-values

## Discussion

Stereotactic irradiation is the method of choice in cases of medically inoperable ES-NSCLC. However, as these are often patients with multiple intercurrent diseases, doubts persist as to which patients are still suitable for curative treatment. Here, we present low early mortality in a real-life cohort and, thus, we consider SABR to be feasible for all medically inoperable patients with reasonable performance status.

Short-term deaths at 6 and 12 months occurred in 4% and 15% of patients, respectively, which is similar to the results from pioneering study RTOG0236 [26], as well as recent studies SPACE [27] and TROG09.02 CHISEL [28]. The median and 3-year OS in our cohort was 35 months and 49%, respectively, which is comparable to large cohorts of peripheral tumors [29].

Apparent local control in 3 years was 90%, what is in agreement with other reports [26, 30]. High local control in our cohort seems to be due to the high prescription dose, as presented by Lee et al. [4] and some others. Regarding the dose-response relationship between local control, OS, and BED, a few reports described an escalated dose in the PTV being associated with a better local control and/or OS [31–34]. These studies suggest that using the convention of prescribing to the 95% isodose line is not ideal for SBRT, as this would lead to a much lower D_max_, which is in agreement with our strategy of a lower isodose line (median 79%). However, BED_max_ only exhibited a trend of better OS prediction and was not significant (Fig. 3).

In our cohort, toxicity was mild (mostly radiological signs of radiation pneumonitis), and no grade 4 and 5 toxicity was detected. Interestingly, we could not confirm poorer results for central tumors, such as due to underdosing with respect to normal tissue tolerance or overdosing organs at risk (OARs). The phase I/II study dealing with central ES-NSCLC (RTOG0813) reported a 7.2% rate of protocol-specified dose-limiting toxicity, including three SBRT-related deaths [35]. In our cohort, a central location was present in only 13% of cases, and we did not use a high dose, such as 55–60 Gy in 5 fractions. Lastly, we used precise image guidance with small CTV-PTV margins.

As expected, AACCI had significant predictive value for OS, but multifactorial analysis indicated a “blanking period”, as only a small difference (3–4%) was found in both the 6- and 12-month survival for low and high AACCI sub-groups. The comorbidity burden was an obvious detrimental factor for OS, but only limited studies have explored how the risk of death among stage I/II NSCLC patients treated with SBRT varies by comorbidity burden [15]. In 2017, Klement et al. [20] described low early mortality and only a 6% difference in the 6-month survival between low/high risk sub-groups, concluding that SBRT should be offered to all patients regardless of their comorbidities unless the performance status prevents accurate SBRT planning and delivery. On the other hand, Baker et al. proposed the Cumulative Illness Rating Scale (CIRS) as a more useful tool than CCI to predict short-term life expectancy [12]. Recently, the same group developed a nomogram that can provide individual survival predictions and assist with treatment decision-making [16]. This is not in agreement with our results.

Patient age was not included as a covariate but was scored in the AACCI. Advanced age is commonly perceived as an adverse prognostic feature, and elderly patients with lung cancer may be less likely to receive active treatment than younger patients. Paradoxically, in our study, the variable age under 78 years was associated with worse chance of surviving than older patients due to the higher burden of comorbidity in these patients. On the other hand, the 1-year mortality rate in our cohort was slightly higher than recently published. In 2020, early deaths in a retrospective study of patients older than 75 years were reported to be 7% in 1 year [36]. Similarly, in patients older than 80 years, the 1-year mortality was 11% [37]. These results indicate longer survival than in our cohort, which may be due to selection bias in the indication of curative treatment and insufficient staging, as PET/CT was not available for all our patients.

Despite the known impact of COPD severity on OS [38], FEV1%, as a variable of pulmonary function, was not associated with early mortality. Given the demonstrated safety and efficiency of SBRT for these patients [39], age and COPD severity should not preclude treatment with curative intent. In a comparison with older studies [10, 40], we could not confirm T stage as a strong prognostic factor for mortality.

BED tended to influence survival. The predictive value of a BED > 100 has been known for a long time [41], and virtually all patients in our cohort fulfilled this condition. Therefore, it is rather a mediated dependence where the physician prescribes a lower dose in patients with expected lower functional reserves.

The strengths of this study include the high number of patients and consistent treatment protocol with precise image guidance and high delivered dose over a long period of time. A limitation of this study is that approximately one-half of the patients (52%) did not have histological verification, mainly due to poor lung function and a risk of serious complications. Another limitation is the retrospective nature and difficulty harvesting patient data. Finally, we were unable to report the cause of death because this information was not available for the majority of patients.

## Conclusion

AACCI is a significant factor for survival, but its prediction value disappears by 1 year. OS was clearly higher than would have been expected after best supportive care only, but we were unable to predict early mortality in this population. Thus, SABR with precise image guidance is feasible for all medically inoperable patients with reasonable performance status after repeated evaluation.

## Data Availability

The datasets used during the current study are available from the corresponding author on reasonable request.

## Abbreviations

AACCI: Age-adjusted Charlson comorbidity index
BED: Biologically effective dose
CCI: Charlson Comorbidity Index
CI: Confidence interval
CIRS: Cumulative Illness Rating Score
COPD: Chronic obstructive pulmonary disease
CT: Computed tomography
ES-NSCLC: Inoperable early-stage non-small cell lung cancer
FEV1: Forced expiratory volume in 1 s
GOLD: Global Initiative for Chronic Obstructive Lung Disease
GTV: Gross tumor volume
HR: Hazard ratio
ITV: Internal target volume
MDT: Multidisciplinary team
OS: Overall survival
PET: Positron emission tomography
PS: Performance status
PTV: Planning target volume
SABR: Stereotactic ablative radiotherapy
SBRT: Stereotactic body radiation therapy
SUVmax: maximum standardized uptake value
